# Doctors’ perception on the ethical use of AI-enabled clinical decision support systems for antibiotic prescribing recommendations in Singapore

**DOI:** 10.3389/fpubh.2024.1420032

**Published:** 2024-07-01

**Authors:** Zhilian Huang, Hannah Yee-Fen Lim, Jing Teng Ow, Shirley Hsiao-Li Sun, Angela Chow

**Affiliations:** ^1^Department of Preventive and Population Medicine, Office of Clinical Epidemiology, Analytics, and Knowledge [OCEAN], Tan Tock Seng Hospital, Singapore, Singapore; ^2^Nanyang Business School, Nanyang Technological University, Singapore, Singapore; ^3^School of Social Sciences, Nanyang Technological University, Singapore, Singapore; ^4^Lee Kong Chian School of Medicine, Nanyang Technological University, Singapore, Singapore; ^5^Saw Swee Hock School of Public Health, National University of Singapore, Singapore, Singapore

**Keywords:** artificial intelligence, antibiotic clinical decision support, clinical vignette, qualitative study, ethics—clinical

## Abstract

**Objectives:**

The increased utilization of Artificial intelligence (AI) in healthcare changes practice and introduces ethical implications for AI adoption in medicine. We assess medical doctors’ ethical stance in situations that arise in adopting an AI-enabled Clinical Decision Support System (AI-CDSS) for antibiotic prescribing decision support in a healthcare institution in Singapore.

**Methods:**

We conducted in-depth interviews with 30 doctors of varying medical specialties and designations between October 2022 and January 2023. Our interview guide was anchored on the four pillars of medical ethics. We used clinical vignettes with the following hypothetical scenarios: (1) Using an antibiotic AI-enabled CDSS’s recommendations for a tourist, (2) Uncertainty about the AI-CDSS’s recommendation of a narrow-spectrum antibiotic vs. concerns about antimicrobial resistance, (3) Patient refusing the “best treatment” recommended by the AI-CDSS, (4) Data breach.

**Results:**

More than half of the participants only realized that the AI-enabled CDSS could have misrepresented non-local populations after being probed to think about the AI-CDSS’s data source. Regarding prescribing a broad- or narrow-spectrum antibiotic, most participants preferred to exercise their clinical judgment over the AI-enabled CDSS’s recommendations in their patients’ best interest. Two-thirds of participants prioritized beneficence over patient autonomy by convincing patients who refused the best practice treatment to accept it. Many were unaware of the implications of data breaches.

**Conclusion:**

The current position on the legal liability concerning the use of AI-enabled CDSS is unclear in relation to doctors, hospitals and CDSS providers. Having a comprehensive ethical legal and regulatory framework, perceived organizational support, and adequate knowledge of AI and ethics are essential for successfully implementing AI in healthcare.

## 1 Introduction

Artificial intelligence (AI) in medicine refers to the use of techniques, such as machine learning and natural language processing, to generate insights that can improve patient health outcomes (1). The rise of digitalization in the past decade grants AI immense potential to revolutionize healthcare systems (2). AI offers novel solutions to many of the challenges faced by healthcare systems today, such as improving diagnostic accuracy (3–5), optimizing workflows (6, 7), and saving time. AI has been utilized in healthcare to improve clinical decision support systems (CDSSs). For example, Juang et al. (5) found that their AI-enabled CDSS outperformed the traditional rule-based CDSS in holistic healthcare prediction for inpatients, demonstrating an improvement in sensitivity from 26.44 to 80.84% and specificity from 99.23 to 99.95%.

Despite the potential of AI-enabled CDSSs in improving clinical outcomes, the ethical considerations surrounding the use of such tools in healthcare has hampered their adoption (8). As AI is increasingly used in healthcare, it shifts the current paradigm of clinical decision making and introduces new ethical implications into the patient-doctor relationship. For instance, AI systems have been shown to contain algorithmic biases that perpetuate human prejudices and disproportionately affect marginalized groups in society (9). Algorithmic biases can stem from human error, where AI developers subconsciously incorporate their personal views and perceptions during data handling (10). Alternatively, algorithmic biases can also stem from using a training dataset that insufficiently represents a minority group, resulting in unfair treatment (10). AI-enabled CDSSs also diminishes a patient’s autonomy when the recommendations do not account for the patient’s personality and preferences (11, 12). For example, an AI-enabled CDSS might be focused on providing the best treatment options that can prolong a patient’s life, but the patient may wish for pain reduction instead. In addition, information crucial for a sound medical judgment might not be incorporated into the AI-enabled CDSS, leading to suboptimal care (11).

These ethical issues are far from theoretical. A 2019 study showed that a widely used prediction algorithm in the US had incorrectly concluded that Black patients are healthier than White patients despite being equally sick because the system had used healthcare spending, instead of health condition, as a proxy for healthcare needs (13). Similarly, in the dermatological field, AI algorithms tended to underperform on images from dark-skinned individuals, as datasets from predominantly fair-skinned individuals were used to train these models (14). Therefore, well-intended implementations of AI-based tools may inadvertently result in poor medical outcomes and injustice if potential ethical issues are overlooked.

Doctors should be cognizant of the potential ethical issues arising from adopting AI-enabled CDSSs as the key decision-makers of their patients’ treatment plans. AI-based decision support systems often fail to consider nuances such as patients’ personality, preferences, and life situations. Doctors must also be aware of the ethical implications that accompany the adoption of AI-enabled CDSSs to mitigate harm and ensure responsible usage. In recent years, the growing literature highlights the need for doctors to understand both the technical limitations and ethical implications of AI decision support systems to trust and integrate the system into daily medical care (11, 15, 16). Therefore, while extensive literature has focused on evaluating the reliability and validity of AI-enabled CDSSs, concentrating solely on the capabilities of AI is insufficient in driving their adoption in healthcare.

Given the inevitable use of AI in healthcare, studying doctors’ understanding and perception of ethical issues that may arise from using AI-based CDSSs would be crucial for the successful implementation of such tools in clinical practice. Furthermore, assessing doctors’ situational knowledge of AI-related ethical dilemmas is a more accurate representation of doctors’ knowledge gaps in ethics in AI. The interplay between ethics, policies and the art of practicing medicine can significantly influence the acceptance and implementation of AI-based tools in healthcare. As rule-based CDSSs for antibiotic prescribing are well established in Singapore’s public healthcare institutions, we chose to use antibiotic prescribing decision support as the context to assess medical doctors’ ethical stance in situations that arise in adopting an AI-enabled CDSS. Our study is also timely as Singapore commits to deepening the use of AI for the public good to uplift and empower people and businesses (17).

## 2 Methods

### 2.1 Setting

We conducted the study in Singapore’s second-largest acute care public hospital, Tan Tock Seng Hospital (TTSH). The hospital operates 2000 beds and serves approximately 1.4 million Singapore residents living within Central Singapore. Although TTSH mainly serves the local population, non-locals can also seek care at the hospital. TTSH implemented a rule-based CDSS—Antimicrobial Resistance Utilization and Surveillance Control (ARUS-C)—to guide doctors in antibiotic selection and dosing in the 2010s (18). All doctors working at TTSH would have used ARUS-C at some point if they had prescribed antibiotics.

Singapore launched the National AI strategy in 2019 as part of the Smart Nation initiative to harness AI’s digital capabilities to improve the lives of Singaporeans (19). Singapore is well-positioned to delve into AI as it has a highly educated workforce and a vibrant technology start-up ecosystem (19). The upgrade of the electronic medical record system in TTSH in 2023 opens up possibilities for incorporating AI into daily clinical practice.

### 2.2 Participants

We conducted in-depth interviews (IDIs) with 30 doctors working in the inpatient setting in TTSH between October 2022 and January 2023. Doctors of various seniority were purposively sampled from a mix of medical and surgical specialties to ensure maximum variation.

### 2.3 Development of the interview guide

We anchored our semi-structured IDI guide on the four pillars of medical ethics—Autonomy, Beneficence, Non-Maleficence, and Justice (20). After conducting a comprehensive review of the literature on possible ethical issues that may arise from using an AI-enabled CDSS, we created hypothetical scenarios of medical dilemmas to understand doctors’ stances when faced with these issues. Table 1 shows some examples of the ethical dilemmas posed to doctors. The protagonist in the vignette (named Max) is a surgeon working at Goodwell Hospital. The hospital has seen an increasing number of inpatients with antimicrobial-resistant infections. Hence, it has decided to implement an AI-enabled CDSS, trained on its database of previously admitted patients, to tackle this problem. We also explored the medico-legal aspects of using an AI-enabled CDSS, and the facilitators of adopting the AI system. We then piloted the interview guide with one junior and two senior doctors who used to work in TTSH and have experience using ARUS-C. Piloting the interviews with these doctors enabled us to check for understanding and flow of the interview guide to refine the guide before conducting the IDIs.

**Table 1:** Ethical dilemmas based on the four pillars of ethics and medico-legal concerns.

Ethics	Scenario	Dilemma
**Justice** (Benefits and cost are distributed equally among all groups)	During Max’s shift, a 65-year-old female tourist from South India was scheduled for a colorectal resection. The surgery was successful, but the patient developed fever and chills 2 days post-surgery and complained of pain at the surgical site. Max suspects the patient has developed a surgical site infection and immediately orders a blood culture. While waiting for the blood culture results, he proceeds to prescribe empirical antibiotics for the patient using the AI-enabled CDSS	Do you think Max should prescribe the empiric antibiotic recommended by the AI tool? Why or why not? Do you have any concern where the data used to generate the recommendation comes from?
**Beneficence** (Doing and promoting good)	The AI-enabled CDSS recommends a narrow-spectrum antibiotic as the best treatment option for the patient. Max thinks that a broad-spectrum antibiotic will better cover for the patient’s infections. However, Max is concerned about the possible antibiotic resistance	What should Max prescribe? What do you think would be in the best interest of the patient, following the best treatment option suggested by the AI-enabled CDSS or your own clinical judgment?
**Autonomy** (Freedom to make choices)	The AI-enabled CDSS recommended an antibiotic that optimizes treatment for the patient’s infection. However, the patient does not wish to continue the antibiotic course due to the fear of side effects. The patient refuses the treatment despite Max’s explanation	What should Max do? What are your thoughts about the AI-enabled CDSS’s influence on Max’s professional autonomy?
**Non-Maleficence** (Do no harm)	De-identified data was given to a third-party company to build Goodwell hospital’s AI-enabled CDSS. Due to the impracticality of seeking individual consent for the large amount of data involved and the lack of requirement under the personal data protection law to do so for de-identified data, a waiver of documentation of consent was approved by the institutional ethics review board. Therefore, consent was not taken from the patients. Four months later, the third-party company that developed the AI-enabled CDSS for Goodwell hospital informs the hospital that the database has been hacked and there was a data breach	What are you most concerned about after this data breach? As Max is a member of the data protection workgroup, what should Max do to mitigate this problem?
**Medico-legal**	Max proceeds to prescribe the antibiotic recommended by the AI-enabled CDSS. The blood culture results showed that the patient was carrying Bacteria A, which was resistant to the narrow-spectrum antibiotic recommended by the AI-enabled CDSS. This type of bacteria is prevalent in India, but uncommon in Singapore	Assuming the patient deteriorates due to Max following the recommendations of the AI-enabled CDSS, do you think Max should be held liable? Why or why not?

### 2.4 In-depth interviews

We conducted the IDIs face-to-face or via video conferencing (i.e., Zoom). Each IDI comprises a note-taker and an interviewer. The note-taker progressively filled a matrix framework with interview notes to assess for data saturation. We first assessed doctors’ familiarity with an AI-enabled CDSS and their perception of how such a future-state system can support them in antibiotic prescribing decisions. Next, we introduced the vignette, walked participants through various ethical dilemmas, and solicited their views from the protagonist’s perspective. We also presented a mock-up of an AI-enabled CDSS to help participants visualize the potential capabilities of such a system as we walk them through the vignette.

### 2.5 Analysis

All IDIs were transcribed verbatim and reviewed for accuracy by a third study team member. We utilized the applied thematic approach, a widely recognized qualitative analysis method in public health studies, to analyze the data (21).

At the initial analysis stage, an independent interview transcript coder familiarized himself with all the transcripts and organized them in NVivo (22) according to the participants’ attributes, such as their professional designation (senior or junior doctors) and their clinical specialty (medical or surgical). Subsequently, the coder identified and compared narratives of participants’ perceptions of the various ethical scenarios, their ethical considerations in antibiotic prescribing decisions, and the factors influencing their decision-making with an AI-enabled CDSS.

A preliminary codebook was developed according to the research objectives after coding the first 10 transcripts, followed by a review of the thematic analysis by the primary research team to eliminate bias, ensure comprehensiveness, and refine the preliminary codes. Several meetings were convened between the coder and the primary research team to ensure alignment in interpretation. The thematic codes were derived inductively, discussed, and agreed upon with the primary research team. Emergent themes were subsequently identified, and the analysis was finalized. Data saturation was achieved at the 20th transcript.

## 3 Results

Half of the participants were non-specialist doctors (i.e., medical officers, residents, senior residents/registrars, and resident physicians) and half were specialist doctors (i.e., associate consultants, consultants, and senior consultants). Two-thirds (63.7%) of participants were from a medical specialty (e.g., general medicine, geriatric medicine, cardiology), while the rest were from a surgical specialty. Almost two-thirds (63.3%) of participants were male, and a similar proportion had been practicing medicine for more than 9 years. Two-thirds (66.6%) of participants lacked a prior understanding of the functionalities of AI-CDSSs and their application in making antibiotic prescribing recommendations (Table 2). The summary of participant quotes, organized according to our interview themes, can be found in Supplementary Table S1.

**Table 2:** Demographic characteristics of participants.

Demographic characteristics	No. of participants (*N* = 30)
**Age (in years)**	
Median (min, max)	36 (26, 49)
**Years of practice as a doctor, *n* (%)**	
2–5	6 (20.0%)
6–9	5 (16.7%)
>9	19 (63.3%)
**Sex, *n* (%)**	
Male	19 (63.3%)
Female	11 (36.7%)
**Basic medical education, *n* (%)**	
Singapore	13 (43.3%)
Overseas	17 (56.7%)
**Professional designation, *n* (%)**	
Medical Officers/Residents	9 (30.0%)
Senior Residents/Registrars	2 (6.7%)
Resident Physicians/Senior Resident Physicians/Principal Resident Physicians	4 (13.3%)
Associate Consultants	4 (13.3%)
Consultants	6 (20.0%)
Senior Consultants	5 (16.7%)
**Specialty of practice, *n* (%)**	
General Medicine	6 (20.0%)
General Surgery	4 (13.3%)
Geriatrics Medicine	4 (13.3%)
Cardiology	2 (6.7%)
Urology	2 (6.7%)
Neurosurgery	2 (6.7%)
Orthopedic Surgery	2 (6.7%)
Other medical specialties*	8 (26.7%)
**Prior knowledge about AI-enabled CDSS, *n* (%)**	
Lacked a prior knowledge	20 (66.6%)

*Other medical specialties include Endocrinology (1), Gastroenterology (1), Hematology (1), Medical Oncology (1), Neurology (1), Renal Medicine (1), Respiratory and Critical Care Medicine (1), and Rheumatology, Allergy and Immunology (1).

### 3.1 Perception of AI-enabled CDSSs

Participants had mixed sentiments when asked to describe their perceptions of the utility of an AI-enabled CDSS. Some doctors felt that the AI-enabled CDSS might be useful in providing a more comprehensive analysis of the patients’ treatment needs, particularly when doctors require guidance on the most appropriate treatment for their patients.

*“AI is advantageous because in a sense they can crunch a lot of data quickly. And it can follow a set algorithm quite accurately. So that’s an advantage. It can really bring in all the factors and consider all that and crunch it very quickly to come up with a final decision.”* (Participant 15, Senior, Surgical specialty, 20 years of practice)

*“On the ground, I have seen a lot of multi-resistant organisms, and sometimes I feel that when I am on call, I may not know what the best antibiotic to order is. Given the fact that some of my peers have not been in Infectious Diseases postings, I think it would be a good tool to guide us and our choice of antibiotics.”* (Participant 03, Junior, Medical specialty, 5 years of practice)

However, some doctors were skeptical of the applicability of the trained AI system on real-world patients as the system might not have been trained on a comprehensive data set. Hence, they could not fully trust the recommendations provided by the AI-enabled CDSS. A few doctors also raised concerns that the AI-enabled CDSS might reduce the “human touch” in patient-doctor interactions.

*“I know specific patients, they are unique. So, I am not really sure about the accuracy, even though it was mentioned that the AI CDSS tool is based on the large pool of data and would recommend antibiotics correctly. So, I am not sure about the accuracy when it is applied to real patients.”* (Participant 29, Junior, Medical specialty, 14 years of practice)

*“Then where is the humanistic aspect of medicine, that is still being practiced? All these things are just going to take away the time with our patients, right? I feel we cannot take away the human touch. I think a lot of patients get better just by you sitting down and talking to them.”* (Participant 22, Senior, Medical specialty, 19 years of practice)

### 3.2 Justice

The principle of Justice relates to the fair distribution of burdens and benefits of new treatments among all groups. In our case, the AI-enabled CDSS trained with local data may not make appropriate antibiotic prescribing recommendations for the patient, a tourist from South India, if the organism causing the infection is not prevalent in Singapore. Participants demonstrated varying levels of awareness on this issue and differing perceptions of whether they should follow the recommendations of the AI-enabled CDSS.

#### 3.2.1 AI-enabled CDSSs trained on local datasets may underrepresent non-local populations

Almost half of participants [14 (47%)] only realized the ethical dilemma after the interviewer probed them to think about the AI-enabled CDSS’s data source. These participants eventually recognized the importance of considering the patient’s background when using an AI-enabled CDSS trained solely on local data which comprised predominantly the local population. A few participants [6 (20%)] did not address the ethical dilemma in the vignette.

*“How long the patient has been here will predispose him to the kind of organism that he probably has or picked up. Within the population, we get certain commensal organisms, which will be different from somebody from another regional country. So, I would say that the time spent in Singapore [should be considered].”* (Participant 23, Junior, Surgical specialty, 7 years of practice)

Participants who would not follow the recommendations made by the AI-enabled CDSS were concerned about the possibility of different strains of bacteria causing the tourist’s infection. Some of them mentioned that they might consider the AI-enabled CDSS’s recommendations but will be cautious in prescribing the recommended antibiotic.

*“Their antibiotic profile, the bacteria profile and the susceptibility are completely different. So, the AI in this case may not necessarily be able to give you a “flash solution” that is specifically tailored, because it will not be able to learn based on a completely new and sudden dataset. It can only learn from what it has known before. So that’s the difference between AI and a physician.”* (Participant 02, Junior, Surgical specialty, 7 years of practice)

One participant trusted that the hospital would have ensured the AI-enabled CDSS was co-developed with infectious diseases specialists and validated to make accurate recommendations.

*“So, if you told me that this software was designed by our [infectious diseases] physicians, I have an inherent trust in their ability and will be satisfied that they would have taken into account the variables that need to be taken into account.”* (Participant 14, Senior, Medical specialty, 8 years of practice)

### 3.3 Beneficence

The principle of Beneficence is the obligation of doctors to act for the benefit of the patient. Participants were asked to choose between a broad-spectrum antibiotic with better coverage for an unknown infection or the AI-recommended antibiotic tailored to the patient. While participants acknowledge the potential of the AI-enabled CDSS in improving patient outcomes, they preferred to exercise their clinical judgment over the AI’s recommendations in the best interest of their patients.

#### 3.3.1 Participants acknowledged the AI-enabled CDSS’s potential in improving patient outcomes

Some doctors felt that the AI-enabled CDSS could provide more accurate recommendations by providing validated recommendations and limiting human error.

*“I would want to reduce prescription errors as well - I think if there was a better choice and the tool helps me with better choice, then that is something that I would certainly consider.”* (Participant 04, Junior, Medical specialty, 2 years of practice)

#### 3.3.2 Participants considered exercising clinical judgment over the AI-enabled CDSS’s recommendations as acting in patients’ best interest

Half [15 (50%)] of our participants would choose to prescribe a broad-spectrum antibiotic, as they felt that an AI-enabled CDSS trained solely on local data may not provide reliable recommendations. Those working in specialties with immunocompromised patients (e.g., Hematology) would also tend to prescribe broad-spectrum antibiotics as they felt that “playing safe” is acting in their patients’ best interest.

*“For Haematology, to be honest, our patients are mostly neutropenic, so for us we would like to offer all possible microbiology there is and we are not comfortable with downgrading to an antibiotic which is not broad spectrum.”* (Participant 05, Junior, Medical specialty, 18 years of practice)

One-third [10 (33.3%)] of participants would follow the AI-enabled CDSS’s recommendation to prescribe the tailored antibiotic as they felt that the AI-enabled CDSS should have considered many possibilities, and a narrow-spectrum antibiotic would be appropriate. Participants who did not specify a decision on Max’s behalf mentioned the lack of patient information in the vignette.

*“Because there is already a surgical site infection there, I presume that [Max] has already input the details [in the AI-enabled CDSS]. Then this AI-enabled CDS tool will help him to prescribe the proper antibiotics. The case is quite outright, very straightforward so I think he should follow the whatever recommendation the AI gives.”* (Participant 05, Junior, Medical specialty, 18 years of practice)

### 3.4 Autonomy

Patient autonomy is the idea that individual patients should have the freedom to make choices about their lives, including medical matters, while physician autonomy is the freedom to determine both the conditions of practice and the care delivered with the principal goal that care decisions are aimed at promoting the patient’s well-being.

#### 3.4.1 Doctors do not feel that AI will take away their autonomy

Most participants felt that adopting an AI-enabled CDSS would not erode their autonomy in making the final decision for their patients, as they are liable for medical malpractice arising from their care. In contrast, a few participants felt that over-reliance on the tool may erode or prevent doctors from sharpening their clinical acumen.

*“I feel that [AI] wouldn’t affect practicing professional autonomy, because the AI is just a tool to determine, by evidence, which is the best choice for the patient.”* (Participant 09, Junior, Medical specialty, 2 years of practice)

*“[The AI-enabled CDSS] will increase the knowledge of doctors [who are] using the tool, but it may [also] diminish clinical judgement because whenever we rely on [the AI-enabled CDSS], it’s like a muscle you don’t exercise. After a while, the clinical judgement may go away.”* (Participant 24, Junior, Medical specialty, 2 years of practice)

#### 3.4.2 Doctors prioritize beneficence over patient autonomy

When we asked participants what they would do if the patient refuses (the most appropriate) treatment for their condition for fear of side effects, many participants mentioned that they would try to convince the patient to follow the “best treatment.” Only 10 (33%) suggested looking for alternative treatments.

“*Well, you should document the process, but you should convince the patient to take the antibiotic in their best interest. It’s important to find out where the patient is coming from. So, you know, what is the underlying - sort of agenda. What is the fear? What is the nature of that fear and the reason behind it? Can that be addressed in a sympathetic way? And, you know, just convince the patient to have the antibiotic.*” (Participant 21, Senior, Medical specialty, 11 years of practice)

*“I think we have to first find out what the patient is able to accept, because all antibiotics come with side effects, right? She knows that she has an infection. So, if she knows that she needs to be treated but she just doesn’t know which antibiotics, then we can ask Max to choose the one with the purported least side effects to assure her.”* (Participant 22, Senior, Medical specialty, 19 years of practice)

### 3.5 Non-maleficence

The principle of Non-maleficence is the obligation of a doctor to do no harm to the patient. In our vignette, we assessed participants’ stance on data breaches due to lapses in data protection. While some participants expressed concerns about the loss of patient confidentiality and the hospital’s reputation, others mentioned that data protection is not in the scope of a doctor’s work.

#### 3.5.1 Doctors do not recognize the implications of data breaches

When asked how Max, as a member of the data protection group, should mitigate the data breach problem, some participants mentioned that it is outside their scope of work. Hence, they are unsure of the steps to mitigate patient data breaches as they do not see themselves playing that role.

*“I'm not a software engineer, I'm not an IT specialist. I do not know. So, what I've been taught about addressing data is that I need to take care of the information I have, patients’ information is not leaked, and carry on doing what I used to do.”* (Participant 26, Senior, Medical specialty, 21 years of practice)

#### 3.5.2 Concerns about the loss of patient confidentiality and hospital’s reputation

Many doctors were not skilled in the laws and regulation of data protection. As the data given to the third-party company was already de-identified, the data does not constitute personal data under the legislation in Singapore and around the world. As far as the Singapore Personal Data Protection Commission is concerned, de-identified data, where the data controller does not hold the key, is anonymized data and is no longer personal data. Two senior doctors, however, mentioned that there is always a risk of re-identifying granular data. Some mentioned that data breach incidents may cause patients to lose confidence in the hospital.

*“If you have deidentified data, then the leaking of the data will not impact the patients negatively. So, I don’t think there are any big issues, to be honest.”* (Participant 07, Senior, Surgical specialty, 12 years of practice)

*“Another thing is that, when such things happen, I would think that probably there has to be some form of… you have to inform the patients whose data has been leaked. It will cause a loss of confidence, in the healthcare system, and that probably has multiple adverse outcomes.”* (Participant 11, Junior, Surgical specialty, 4 years of practice)

### 3.6 Medico-legal

Several participants were hesitant to fully trust the AI-enabled CDSS due to concerns about the ambiguity of frameworks to guide and the lack of regulation to protect doctors in adopting AI tools in medicine.

#### 3.6.1 Doctors should bear the liability of patient deterioration even if the recommendations were from an AI-enabled CDSS

When asked who should be liable for patient deterioration due to the doctor following the recommendations of an antibiotic-prescribing AI-enabled CDSS, many [22 (73%)] participants felt that the liability falls on the final decision maker (i.e., the doctor). One doctor felt that the hospital should share the liability with the doctor if the doctor abides by the hospital’s policy. A few other doctors were cautious about attributing medical liability to any party as medical malpractice cases tend to be situational.

*“I mean at the end of the day the decision falls onto the doctor, right? So, whoever prescribes [the antibiotic]. It’s not ideal but that’s the way it is. You can’t blame a machine.”* (Participant 10, Senior, Medical specialty, 16 years of practice)

*“The hospital is responsible for this rather than the doctor personally. Because the doctor has been advised by the hospital to use the tool and he's gone by the hospital's policy. So, I don't think the doctor is individually held responsible, it’s a collective responsibility.”* (Participant 26, Senior, Medical specialty, 21 years of practice)

“*In terms of whether Max is liable, I think not entirely. He has followed what is recommended. Of course, whether is someone is at fault, I guess it’s up to the judiciary or a tribunal to settle, to ascertain culpability and liability. I mean, who has implemented it and how robust it was tested before it was rolled out, you know. I wouldn’t say Max is at fault or think he is. He followed what was recommended, the tool that was recommended for him to use and he has trust in the system, the system has vetted through the [AI-enabled CDSS], that it is safe to use.”* (Participant 28, Junior, Medical specialty, 18 years of practice)

### 3.7 Trust in AI systems

Trusting the AI system and its decisions facilitates the adoption of AI-enabled CDSS. We asked participants about the factors that instill confidence in adopting an AI-enabled CDSS after the vignette. These factors include allowing doctors to retain their professional autonomy, ensuring the system is validated and up-to-date, accounting for non-local populations, and improving patient outcomes.

*“I guess if there have been trials in other countries, other institutions, other hospitals that have used it. Similar program and what their results are, obviously stats, and numbers comparing clinicians not using it versus the AI, the drug resistance rates, the number of adverse events [resulting from] prescribing with or without, the narrow spectrum versus broad spectrum. I think trials may need to be done, or pilot studies may to be done to demonstrate that. Would obviously give more confidence to the clinician.”* (Participant 17, Junior, Surgical specialty, 7 years of practice)

## 4 Discussion

Our study explored doctors’ stance in ethical dilemmas arising from adopting an AI-enabled CDSS for making antibiotic-prescribing decisions. Visuals and a structured vignette helped participants grasp the concept of AI-enabled CDSS better (16) and enabled the interviewers to engage in meaningful conversations with the participants. Previous research has examined possible ethical issues that may arise from applying AI-driven capabilities in healthcare, but few studies have considered the views of on-the-ground practitioners. Our study contributes to a deeper understanding of the ethical considerations of applying AI-based tools in healthcare by soliciting doctors’ views on ethical dilemmas arising from adopting an AI-enabled CDSS. Doctors’ perspectives on AI-based CDSS are essential as they are the key stakeholders in determining the success of AI implementation in the clinical setting. The interview also allowed us to explore the interplay between doctors’ judgment and trust in an AI-enabled CDSS’s reliability from the ethics perspective.

Although 80% of participants eventually recognized that the AI-enabled CDSS’s recommendations might be biased against the tourist, almost half of respondents only recognized the issue after probing, and 20% remained unaware of the ethical dilemma posed to them. The AI-enabled CDSS should ideally provide more “precise” treatments to the patient, but not all patients will benefit equally from the AI-enabled CDSS, as “injustice” may unintentionally be introduced to non-local populations. In this case, the exclusion of non-local profiles resulted in a bias toward the tourist, and doctors who did not realize the AI-enabled CDSS’s bias could have unintentionally propagated the bias toward the patient. Implicit and unconscious biases have always existed in human decisions, and basing AI decisions on past human decisions may propagate these biases (23). Therefore, being cognizant of possible biases when introducing a new technology is essential in preventing well-meaning intentions from introducing or amplifying unintended biases.

Half of the participants chose to prescribe a broad-spectrum antibiotic despite knowing that the narrow-spectrum antibiotic recommended by the AI-enabled CDSS would help to slow antimicrobial resistance. Doctors would hesitate to accept the AI-enabled CDSS’s recommendations if the recommendations deviated from their standard care decisions. This hesitancy possibly stems from a lack of exemption from legal liability arising from accepting the recommendations of a “future state” decision support tool (24). Doctors have less incentive to accept non-standard care recommendations as it does not absolve them from malpractice litigations. On the contrary, following standard care recommendations would shield doctors from “unconventional” decisions that increase their legal liability. In addition, the small number of participants who chose to prescribe the broad-spectrum antibiotics valued reducing participants’ suffering above optimizing patients’ treatment outcomes.

Two-thirds of participants would immediately attempt to convince the patient to follow the best treatment recommended by the AI-enabled CDSS, when the patient refuses treatment due to fear of side effects. In Singapore, medical paternalism has been rooted in the healthcare sector as patients perceived doctors as being educated and better informed of their health conditions (25). In return, patients highly trust doctors in directing their treatment. The doctors’ perception of the principle of beneficence is often to act in patients’ best interests, which in our case was convincing the patient to accept the perceived best treatment. The introduction of AI-enabled CDSS may exacerbate paternalism with the doctor convincing the patient to accept the perceived ‘more precise’ antibiotic treatment recommended by the AI-enabled CDSS for the patient. Hence, doctors should be mindful of the patient’s wishes, with the advent of AI-enabled CDSSs.

Many doctors are not well-educated on the legal position of data breaches. Although a few doctors recognized that data breaches may negatively impact the hospital’s reputation and diminish patients’ trust in the health system (26), most stated that it was beyond their job scope to mitigate implications of data breaches. However, some doctors recognized that patients’ distrust in the hospital’s ability to safeguard data might cause them to withhold important information related to their medical condition, which may in turn harm the patient, even though the data was already deidentified. Since legal liability for data breaches may possibly fall on the hospital as well as the company developing the AI-enabled CDSS, clearer policies and laws on liability are needed for anonymized data for healthcare.

Since most (73%) doctors felt they are liable for medical malpractice if they follow the wrong recommendations made by an AI-enabled CDSS, they will be cautious about fully trusting and adopting an AI system that has not been trialed and validated. Liability will ultimately need to fall on a human as responsibility cannot be attributed to AI systems, which are not moral agents possessing free will (27). Participants’ acknowledgement of the potential benefits of AI while also hesitating to accept its recommendations highlights a gap in technology acceptance and implementation. Doctors may wish to embrace AI’s capabilities in their medical practice but fear the possible ethical and legal implications. This hesitancy underscores the need to equip doctors with the knowledge to navigate ethical and legal situations arising from adopting AI-enabled CDSSs. Providing comprehensive guidelines, training, and resources would be a step forward in ensuring the ethical, responsible use of AI-enabled CDSSs in the medical field.

Trust is essential in facilitating AI adoption. Participants mentioned that retaining the doctor’s professional autonomy, keeping technology systems updated, and seeing positive patient outcomes helps to instill confidence in adopting an AI-enabled CDSS. Other studies have also found that fear of losing the doctor’s autonomy and difficulty integrating AI into existing clinical systems were factors hindering AI adoption among doctors (16, 28).

Although we purposively sampled participants of varying seniority from a range of medical and surgical specialties, limitations exist in our study. First, almost all our participants have experienced using a rule-based antibiotic CDSS (ARUS-C) and were accustomed to environments with fast-changing technological systems. Participants’ readiness for technology change was higher compared with other contexts. Second, our study was conducted in one institution in Singapore, limiting the generalizability of our findings to other healthcare systems with different practices or cultures.

Given the inevitable use of AI in healthcare, it is imperative to expand the medical workforce’s knowledge of AI and the law and ethics governing the use of AI to mitigate legal and ethical situations that may arise from AI adoption in the future. The medical community should, among other things, set up a committee to govern the use of AI in medicine to instill trust in AI adoption among healthcare providers. Healthcare institutions should bear responsibilities and draw up AI governance guidance covering potential legal and ethical issues that may arise.

## 5 Conclusion

In conclusion, few doctors were fully cognizant of the ethical issues with AI in healthcare while most would gravitate decisions toward familiar practice contexts when faced with ethical dilemmas. The lack of understanding of the ethics of AI impedes doctors’ trust and readiness to adopt AI in their daily practice. Therefore, a comprehensive ethical and legal framework, organizational support, and adequate knowledge of laws, AI and its ethics, are essential for the successful implementation of AI in healthcare.

## Data availability statement

The datasets presented in this article are not readily available because the data is transcribed from interview transcripts, which cannot be shared publicly according to the ethics guidelines. Requests to access the datasets should be directed to AC, angela_chow@ttsh.com.sg.

## Ethics statement

The studies involving humans were approved by National Healthcare Group Domain Specific Review Board. NHG DSRB Ref: 2022/00483. The studies were conducted in accordance with the local legislation and institutional requirements. The participants provided their written informed consent to participate in this study.

## Author contributions

ZH: Conceptualization, Methodology, Formal Analysis, Project administration, Data curation, Supervision, Writing - original draft, Writing – review & editing. HL: Funding acquisition, Writing – review & editing. JO: Formal analysis, Data curation, Writing - original draft, Writing – review & editing. SS: Conceptualization, Funding acquisition, Writing – review & editing. AC: Conceptualization, Funding acquisition, Resources, Supervision, Writing – review & editing.
