# Supplementary material for: Doctors’ perception on the ethical use of AI-enabled clinical decision support systems for antibiotic prescribing recommendations in Singapore
Source: Front Public Health. 2024 Jul 1;12:1420032. doi: 10.3389/fpubh.2024.1420032 (PMC11246905; doi:10.3389/fpubh.2024.1420032)
Supplement: Supplementary file 1 [file Table_1.DOCX]

**Supplementary Table 1: Themes and representative quotes from the interviews**

| **Themes** | **Senior physician**  **representative quotes** | **Junior physician**  **representative quotes** |
| --- | --- | --- |
| **Perception of AI-enabled CDSSs** | | |
| Participants had mixed sentiments when asked to describe their perceptions of the utility of an AI-enabled CDSSs | *“AI is advantageous because in a sense they can crunch a lot of data quickly. And it can follow a set algorithm quite accurately. So that’s an advantage. It can really bring in all the factors and consider all that and crunch it very quickly to come up with a final decision.”* **(Participant 15, Senior, Surgical specialty, 20 years of practice)**  *“Then where is the humanistic aspect of medicine, that is still being practiced? All these things are just going to take away the time with our patients, right? I feel we cannot take away the human touch. I think a lot of patients get better just by you sitting down and talking to them.”* **(Participant 22, Senior, Medical specialty, 19 years of practice)** | *“[The AI-enabled CDSS] has the benefit of having a very wide resource to fall back on, that it’s learnt to pick up and recognize patterns from. So maybe that will help in more complex patients.”* **(Participant 23, Junior, Surgical specialty, 7 years of practice)**  *“On the ground, I have seen a lot of multi-resistant organisms, and sometimes I feel that when I am on call, I may not know what is the best antibiotic to order. Given the fact that some of my peers have not been in Infectious Diseases postings, I think it would be a good tool to guide us and our choice of antibiotics.”* **(Participant 03, Junior, Medical specialty, 5 years of practice)**  *“I know specific patients, they are unique. So, I am not really sure about the accuracy, even though it was mentioned that the AI CDSS tool is based on the large pool of data and would recommend antibiotics correctly. So, I am not sure about the accuracy when it is applied to real patients.”* **(Participant 29, Junior, Medical specialty, 14 years of practice)** |
| **Justice**  (Benefits and cost are distributed equally among all groups) | | |
| AI-enabled CDSSs trained on local datasets may underrepresent non-local populations | *“I think the limitation I see here is that, you know, the patient is a foreign patient. So, if the AI-enabled tool takes data from the hospital’s historical records, the presumption would then be the majority of those patients would be local. So, I would need to have the base knowledge of what kinds of- to what extent does a patient’s, say, skin flora influences the particular organism causing the surgical site infection?”* **(Participant 21, Senior, Medical specialty, 11 years of practice)**  *“I will look at it and I will determine. Because this is not a local patient, and I do not have any other history. I do not know if she [has had] any other infections. So, I cannot say, hundred percent, that the infection is a local bug. I would look at the CDS recommendation and decide for myself whether I’ll follow [the recommendation].”* **(Participant 08, Senior, Medical specialty, 17 years of practice)**  *“But it sounds like a validated tool, right? I would assume, I mean, if it’s been validated, I would assume it would be fine.”* **(Participant 10, Senior, Medical specialty, 16 years of practice)**  *“So, if you told me that this software was designed by our [infectious diseases] physicians, I have an inherent trust in their ability and will be satisfied that they would have taken into account the variables that need to be taken into account.”* **(Participant 14, Senior, Medical specialty, 8 years of practice)** | *“Their antibiotic profile, the bacteria profile and the susceptibility are completely different. So, the AI in this case may not necessarily be able to give you a “flash solution” that is specifically tailored, because it will not be able to learn based on a completely new and sudden dataset. It can only learn from what it has known before. So that’s the difference between AI and a physician.”* **(Participant 02, Junior, Surgical specialty, 7 years of practice)**  *“It really depends on what factors the AI will have access to, and whether that will be sufficient to correctly represent the most likely organism. I will say [Max] can use that to guide his decision. [If] he agrees with what the AI will recommend then he can prescribe it.”* **(Participant 24, Junior, Medical specialty, 2 years of practice)**  *“How long the patient has been here will predispose him to the kind of organism that he probably has or picked up. Within the population, we get certain commensal organisms, which will be different from somebody from another regional country. So, I would say that the time spent in Singapore [should be considered].”* **(Participant 23, Junior, Surgical specialty, 7 years of practice)** |
| **Beneficence**  (Doing and promoting good) | | |
| Participants acknowledged the AI-enabled CDSS’s potential in improving patient outcomes | *“So that gives us two things. Number one is the accuracy of the treatment, with probably shortened care and [slowing] further drug resistance. Number two, I feel it may help the junior doctors or the on-call doctors reduce their work in flipping through notes with a risk of missing out some data.”* **(Participant 07, Senior, Surgical specialty, 12 years of practice)**  *“The group of patients we look after are the ones who are immunocompromised and they’re quite ill with multiple comorbidities. They’re at risk of infection [and] the infections are serious. So, we need to make a quick decision about antibiotic therapy for these patients. Should we have this, this will help us in making a decision about narrowing the spectrum.”* **(Participant 26, Senior, Medical specialty, 21 years of practice)** | *“I would want to reduce prescription errors as well- I think if there was a better choice and the tool helps me with better choice, then that is something that I would certainly consider.”* **(Participant 04, Junior, Medical specialty, 2 years of practice)**  *“A lot of my patients are all immune suppressed. We tend to just start broad spectrum [antibiotics], so we may not be the best stewardship towards antimicrobial resistance. This thing will probably help us to be better and allow us to have more efficacious, targeted treatment for the patient.”* **(Participant 20, Junior, Medical specialty, 4 years of practice)** |
| Participants considered exercising clinical judgement over the AI-enabled CDSS’s recommendations as acting in patients’ best interest | *"If I have a patient who has a foot infection, for example, whatever I do for the patient has to be kind of a one shot one kill. I don’t have a second chance for that patient. He needs that leg, for quality of life. If I say, “I want to wait for the correct antibiotics and I’m going to ignore everything else”, it means you don’t care that the patient loses the leg and you don’t care the patient’s life will be affected.”* **(Participant 15, Senior, Surgical specialty, 20 years of practice)**  *“I think [you] should follow your own clinical judgement in conjunction with what is suggested by the CDSS. Yes, it’s AI, all the fancy neural networks and everything else. We assume that it’s comprehensive, but there [are] always variations on how the patient presents. At the end of the day, if we give the wrong antibiotic and it’s not targeting the patient correctly, the patient may suffer if we’re not going broad initially.”* **(Participant 19, Senior, Surgical specialty, 12 years of practice)** | *“Because the patient is a tourist, the infection she is carrying may not be what is commonly studied. The data put in AI is based on the local population, the population that is within the Goodwell hospital, so it may not be reflective of where the patient is from. I think it definitely will affect the way I practice in terms of choosing antibiotics to use.”* **(Participant 03, Junior, Medical specialty, 5 years of practice)**  *“For Haematology, to be honest, our patients are mostly neutropenic, so for us we would like to offer all possible microbiology there is and we are not comfortable with downgrading to an antibiotic which is not broad spectrum.”* **(Participant 05, Junior, Medical specialty, 18 years of practice)** |
| **Autonomy**  (Freedom to make choices) | | |
| Physicians do not feel that AI will take away their autonomy | *“I think that [the AI tool] does not limit what we practise, per se, if we use it as a guide. I think at the end of the day, you have your own clinical judgement. It is up to the physician himself to exercise it.”* **(Participant 19, Senior, Surgical specialty, 20 years of practice)**  *“You’re still autonomous. The [AI] tool is an aid, but the ultimate decision is with the clinician.”* **(Participant 08, Senior, Medical specialty, 17 years of practice)** | *“I feel that [AI] wouldn’t affect practicing professional autonomy, because the AI is just a tool to determine, by evidence, which is the best choice for the patient.”* **(Participant 09, Junior, Medical specialty, 2 years of practice)**  *“I think you still have total autonomy. [The AI-enabled CDSS] just gives you options.”* **(Participant 17, Junior, Surgical specialty, 7 years of practice)**  *“[The AI-enabled CDSS] will increase the knowledge of doctors [who are] using the tool, but it may [also] diminish clinical judgement because whenever we rely on [the AI-enabled CDSS], it’s like a muscle you don’t exercise. After a while, the clinical judgement may go away.”* **(Participant 24, Junior, Medical specialty, 2 years of practice)** |
| Physicians prioritize beneficence over patient autonomy | *“Well, you should document the process, but you should convince the patient to take the antibiotic in their best interest. It’s important to find out where the patient is coming from. So, you know, what is the underlying- sort of agenda. What is the fear? What is the nature of that fear and the reason behind it? Can that be addressed in a sympathetic way? And, you know, just convince the patient to have the antibiotic.”* **(Participant 21, Senior, Medical specialty, 11 years of practice)**  *“I think we have to first find out what the patient is able to accept, because all antibiotics come with side effects, right? She knows that she has an infection. So, if she knows that she needs to be treated but she just doesn’t know which antibiotics, then we can ask Max to choose the one with the purported least side effects to assure her.”* **(Participant 22, Senior, Medical specialty, 19 years of practice)** | *“[Max] should actually go back to the patient and really convince [her], if the patient has mental capacity. Sometimes I would also get the family members as well. So, call the mother, father or siblings, or the spokesperson, to just help us with convincing the patient”* **(Participant 05, Junior, Medical specialty, 18 years of practice)**  *“If [the patient] has capacity and is able to understand the risk of refusing antibiotics, at least we should take into account her own personal beliefs. I mean, we can try our best to counsel her but at the end of the day, if she is able to make her own decisions, we should not override her or force antibiotics on her.”* **(Participant 04, Junior, Medical specialty, 2 years of practice)** |
| **Non-Maleficence**  (Do no harm) | | |
| Physicians do not recognize the implications of data breaches | *“I'm not a software engineer, I'm not an IT specialist. I do not know. So, what I've been taught about addressing data is that I need to take care of the information I have, patients’ information is not leaked, and carry on doing what I used to do.”* **(Participant 26, Senior, Medical specialty, 21 years of practice)**  *“I mean if it happens, it happens. We have to look at the infrastructure and see how we can prevent it. [That’s] on the IT side. I think on the physicians’ side, we- we don't really think too much or bother too much about all these things.”* **(Participant 27, Senior, Medical specialty, 9 years of practice)** | *“Isn’t the breach due to the system [being] hacked? We can’t really do much about that apart from robust IT services, right? As an individual you can’t really do much about it.”* **(Participant 17, Junior, Surgical specialty, 7 years of practice)**  *“I don’t know. Firewall? I don’t know. This is too technical for me.”* **(Participant 06, Junior, Surgical specialty, 14 years of practice)** |
| Concerns about the loss of patient confidentiality and hospital’s reputation | *“I think it affects me in the sense that patients who then read about it may feel uncomfortable about coming to seek medical [care] here. They will have that kind of sentiment, that how do I know my data that you are storing here [is safe]. It just basically generates mistrust.”* **(Participant 16, Senior, Surgical specialty, 25 years of practice)**  *“I mean he can say it’s deidentified, but I’m still concerned because that information is now being leaked. People know about the patients being treated at our hospital. So, I’m definitely concerned about patients’ confidentiality.”* **(Participant 12, Senior, Surgical specialty, 10 years of practice)** | *“Another thing is that, when such things happen, I would think that probably there has to be some form of… You have to inform the patients whose data has been leaked. It will cause a loss of confidence, in the healthcare system, and that probably has multiple adverse outcomes.”* **(Participant 11, Junior, Surgical specialty, 4 years of practice)**  *“So, I’m most concerned about sensitive data. Most of it will be sensitive data, like infectious diseases [such as] HIV, sexually transmitted diseases. Especially if it’s released to the open public, that can create a lot of stigma for existing patients. Even routine chronic diseases, that can be used to exploit. It really depends on whether [the deidentification] can be reversed.”* **(Participant 24, Junior, Medical specialty, 2 years of practice)** |
| **Medico-legal** | | |
| Physicians should bear the liability of patient deterioration even if the recommendations were from an AI-enabled CDSS | *“I mean at the end of the day the decision falls onto the doctor, right? So, whoever prescribes [the antibiotic]. It’s not ideal but that’s the way it is. You can’t blame a machine.”* **(Participant 10, Senior, Medical specialty, 16 years of practice)**  *“The hospital is responsible for this rather than the doctor personally. Because the doctor has been advised to, by the hospital, to use the tool and he’s gone by the hospital’s policy. So, I don't think the doctor is individually held responsible, it’s a collective responsibility.”* **(Participant 26, Senior, Medical specialty, 21 years of practice)** | *“It is the clinician who makes the decision at the end of the day. [The AI tool] is just another tool. It’s just like another other reference tool that we use. So, if the decision is made, at the end of the day, by the physician in-charge, then that’s where the ultimate responsibility lies.”* **(Participant 13, Junior, Medical specialty, 7 years of practice)**  *“In terms of whether Max is liable, I think not entirely. He has followed what is recommended. Of course, whether is someone is at fault, I guess it’s up to the judiciary or a tribunal to settle, to ascertain culpability and liability.”* **(Participant 28, Junior, Medical specialty, 18 years of practice)** |
| **Trust in AI systems** | | |
| Factors that instil confidence in adopting an AI-enabled CDSS | *“I think firstly, you know, getting the stakeholders, all of the clinicians, to kind of communicate the value proposition of it, to demonstrate that it is safe. Then, to also demonstrate how it will facilitate clinical judgement, as opposed to removing the autonomy of the clinician. Then [address] the technical aspect of how [the AI tool] integrates into the current system. The last thing is about the skilling of the clinician, because of the AI tool.”* **(Participant 30, Senior, Medical specialty, 13 years of practice)**  *“Personally, I would like to know, is there any validation that is available for this AI tool? Are there any hard outcomes that [have] been shown? You know that it does not cause additional harm and so on. With those it will be a bit more convincing to use it, because otherwise, it’s almost like what you call ‘cookbook medicine’.”* (**Participant 18, Senior, Medical specialty, 18 years of practice)** | *“I guess if there have been trials in other countries, other institutions, other hospitals that have used it. Similar program and what their results are, obviously stats, and numbers comparing clinicians not using it versus the AI, the drug resistance rates, the number of adverse events [resulting from] prescribing with or without, the narrow spectrum versus broad spectrum. I think trials may need to be done, or pilot studies may to be done to demonstrate that. Would obviously give more confidence to the clinician.”* **(Participant 17, Junior, Surgical specialty, 7 years of practice)**  *“So, I think that it should come with the advisory that the data is not exhaustive, that the clinician should still always use their clinical experience and their own clinical acumen to decide on the best antibiotics. I think this tool should only be as recommendation, definitely not something that you cannot override.”* **(Participant 03, Junior, Medical specialty, 5 years of practice)** |
